# Lack of respect and balance

**DOI:** 10.1192/bjb.2021.119

**Published:** 2022-02

**Authors:** Margaret White

**Affiliations:** ST4 in Intellectual Disability Psychiatry, NHS Lothian, Edinburgh, UK. Email: m_i_white@mac.com

This editorial and current issue of BJPsych Bulletin do nothing to be ‘respectful and balanced’ about issues of trans health. Reprinting the article which caused the controversy in the first place means that it is exposed to a wider audience, and instead of having a counterbalancing view in another article, it has the article by Griffin et al which contains a number of anti-trans talking points. Anything which is supportive of trans people or current best practice standards for trans health is relegated to the letter pages. None of the authors of the two articles are gender identity specialists; they have instead mobilised their credentials in other areas to claim expertise in an area where they have none. The voices of trans people are either absent or denigrated as some kind of online-based groupthink.

Trans health is its own research field, and there are plenty of researchers that the *Bulletin* could have reached out to for a counterbalancing view. Instead, they have amplified anti-trans voices once more, with a sop that those with opposing views could write a letter or propose an article.

I am not seeking to silence debate, and acknowledge that this is a controversial area. However, issues around trans health are treated particularly poorly in the *Bulletin*. Would the *Bulletin* accept having two papers on women's mental health written solely by men who had no expertise in women's mental health, or two papers on ethnic minority mental health written solely by white people who had no expertise in ethnic minority mental health? If not, why is it acceptable for this to happen for trans people?

